# Curcumin mediates oxaliplatin-acquired resistance reversion in colorectal cancer cell lines through modulation of CXC-Chemokine/NF-κB signalling pathway

**DOI:** 10.1038/srep24675

**Published:** 2016-04-19

**Authors:** Vicenç Ruiz de Porras, Sara Bystrup, Anna Martínez-Cardús, Raquel Pluvinet, Lauro Sumoy, Lynne Howells, Mark I. James, Chinenye Iwuji, José Luis Manzano, Laura Layos, Cristina Bugés, Albert Abad, Eva Martínez-Balibrea

**Affiliations:** 1 Health Sciences Research Institute of the Germans Trias i Pujol Foundation (IGTP), Can Ruti Campus, Ctra. Can Ruti- Camí de les escoles s/n, 08916, Badalona, Spain; 2 Cancer Epigenetics and Biology Program (PEBC), Bellvitge Biomedical Research Institute (IDIBELL), Gran Via de l’Hospitalet, 199. 08908 L’Hospitalet de Llobregat, Barcelona, Spain; 3 Genomics and Bioinformatics Unit, Institute for Predictive and Personalized Medicine of Cancer (IMPPC), Can Ruti Campus, Ctra. Can Ruti- Camí de les escoles s/n, 08916, Badalona, Spain; 4 Dept Cancer Studies, University of Leicester, Robert Kilpatrick Clinical Sciences Building, Leicester Royal Infirmary, Leicester, LE2 7LX, UK; 5 Medical Oncology Service, Catalan Institute of Oncology (ICO) University Hospital Germans TriasiPujol, Ctra. Can Ruti- Camí de les escoles s/n, 08916, Badalona, Spain; 6 Oncology Unit, Hospital CIMA Sanitas, Barcelona, Catalonia, Spain; 7 Resistance, chemotherapy and predictive biomarkers group. ProCURE (Program against cancer resistance). Catalan Institute of Oncology. Edifici IGTP, Carretera de Can Ruti, Camí de les escoles, s/n, Campus Can Ruti, 08916 Badalona, Spain

## Abstract

Resistance to oxaliplatin (OXA) is a complex process affecting the outcomes of metastatic colorectal cancer (CRC) patients treated with this drug. De-regulation of the NF-κB signalling pathway has been proposed as an important mechanism involved in this phenomenon. Here, we show that NF-κB was hyperactivated in *in vitro* models of OXA-acquired resistance but was attenuated by the addition of Curcumin, a non-toxic NF-κB inhibitor. The concomitant combination of Curcumin + OXA was more effective and synergistic in cell lines with acquired resistance to OXA, leading to the reversion of their resistant phenotype, through the inhibition of the NF-κB signalling cascade. Transcriptomic profiling revealed the up-regulation of three NF-κB-regulated CXC-chemokines, CXCL8, CXCL1 and CXCL2, in the resistant cells that were more efficiently down-regulated after OXA + Curcumin treatment as compared to the sensitive cells. Moreover, CXCL8 and CXCL1 gene silencing made resistant cells more sensitive to OXA through the inhibition of the Akt/NF-κB pathway. High expression of CXCL1 in FFPE samples from explant cultures of CRC patients-derived liver metastases was associated with response to OXA + Curcumin. In conclusion, we suggest that combination of OXA + Curcumin could be an effective treatment, for which CXCL1 could be used as a predictive marker, in CRC patients.

Colorectal Cancer (CRC) is still one of the most frequent causes of cancer-related death worldwide. The 5-year overall survival rate is less than 10% in advanced disease and chemotherapy treatment remains essential for these patients. Thus, despite the availability of targeted therapies against the Epidermal Growth Factor Receptor (EGFR) or the Vascular Endothelial Growth Factor (VEGF), combinations of oxaliplatin (OXA) with fluoropyrimidines (5-fluorouracil or capecitabine) are the most commonly used frontline regimens in the metastatic disease^1^. OXA is a third-generation platinum drug and it is the only platinum analogue that has activity in CRC, in both adjuvant and first-line treatment^2^. OXA cytotoxicity is mainly generated through the formation of platinum-DNA adducts resulting in DNA transcription and replication blockade. As a consequence, several signalling pathways are activated leading to DNA damage repair and/or the activation of cell death programs^3^. However, as with other chemotherapies, its effectiveness is limited by the appearance of drug resistance^4^. Chemoresistance associated with OXA is a complex and multifactorial process in which several mechanisms such as drug influx/efflux modifications, alterations in DNA damage repair, decrease of cell death activation, autocrine survival signalling or high detoxification activity could play a part^5^. Amongst these processes, the Nuclear factor kappa-light-chain-enhancer of activated B cells (NF-κB) has been implicated in the activation of survival pathways following OXA treatment, and may be an important factor in mediating acquired resistance to OXA. NF-κB is a transcription factor that contributes to the progression of CRC by regulating the expression of diverse target genes that are involved in inflammation (e.g. TNFα, IL-1, CXC-chemokines), cell proliferation (e.g. Cyclin D1, COX2, c-myc, IL-6), apoptosis (e.g. XIAP, IAP-1, IAP-2, Survivin, Bcl-2 and Bcl-xl), angiogenesis (e.g. VEGF, IL-8), invasion (e.g. ICAM-1, VCAM-1) and metastasis (e.g. MMP-9)^6^. Constitutive activation of NF-κB has been observed in many solid tumours, including CRC^7^,^8^, and provides a survival mechanism by up-regulating anti-apoptotic genes and thereby representing a major causative factor for drug resistance^9^. Of note, it has been shown that administration of OXA can potentiate NF-κB activity, increasing transcriptional regulation and expression of anti-apoptotic genes^10^. Thus, the inhibition or modulation of NF-κB and its downstream targets has been proposed as an important target for the development of therapeutic approaches against this disease and the resistance to platinum agents^11^. In previous work, we investigated the alteration in gene transcription patterns between sensitive and OXA-acquired resistant human CRC cell lines. Our results led us to hypothesize that the NF-κB signalling pathway was an important contributor in the development of OXA resistance in this model^12^ and that a reasonable strategy for CRC cancer treatment may be the combination of OXA-based chemotherapy with compounds active against NF-κB. One such compound is Curcumin (diferuloylmethane), the major active ingredient of turmeric (*Curcuma longa*). Curcumin, with no discernable toxicity, inhibits the growth of transformed cells^13^,^14^ and also has been shown to suppress initiation, promotion, and progression of colon carcinogenesis in induced rodent models^15^. In the past few years, several studies have demonstrated that Curcumin inhibits NF-κB activity and down-regulates the expression of NF-κB regulated gene products in different cell types at nontoxic concentrations in humans, leading to the suppression of proliferation, cell cycle arrest and induction of apoptosis^16^,^17^. It has also been reported that Curcumin enhances the effect of diverse anti-cancer drugs against CRC, including OXA and 5- fluorouracil, both in *in vitro* and *in vivo* models^18^,^19^,^20^,^21^,^22^,^23^. The anti-tumour activity and safety of Curcumin has been extensively studied in humans, and several clinical trials are on-going in order to evaluate new formulations with greater bioavailability and combinations with conventional chemotherapy^24^,^25^,^26^. Despite its poor systemic bioavailability, Curcumin has been reported to distribute in gastrointestinal tract to a great extent and is independent of systemic availability, demonstrating the potential to prevent and reduce CRC^27^.

The aims of this work were firstly, to demonstrate that the NF-κB pathway was hyper-activated in CRC cells with acquired resistance to OXA and to evaluate whether the combined treatment of Curcumin and OXA could revert this phenotype and secondly, to find one or more predictive markers for the effectiveness of this combination that could be used in the selection of patients with high probability to respond to this treatment.

## Results

### The NF-κB pathway is hyperactivated in CRC cell lines with acquired resistance to OXA

**Figure 1:**
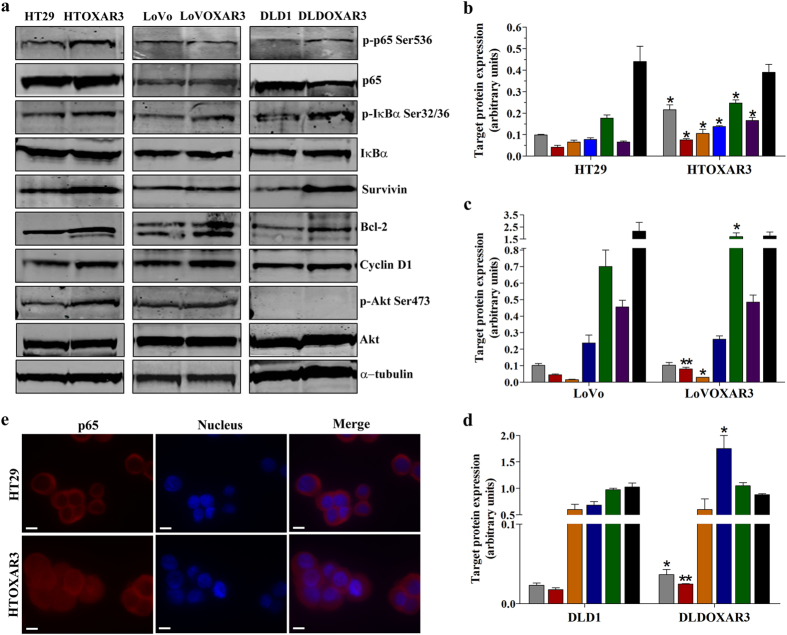
NF-κB pathway activation in CRC cell lines with acquired resistance to OXA. Western blot analysis (**a**) and graphic representation of phosphorylated p65 (p-p65 Ser536, grey bars), phosphorylated IκBα (p-IκBα Ser32/36, red bars), Survivin (orange bars), Bcl-2 (blue bars), Cyclin D1 (green bars), phosphorylated Akt (p-Akt Ser473, purple bars) and IκBα (black bars) basal expression in HT29/HTOXAR3 (**b**), LoVo/LoVOXAR3 (**c**) and DLD1/DLDOXAR3 (**d**) cell lines. Alpha-tubulin was used as endogenous control. (**e**) Representative immunocytochemistry images of the subcellular localization of p65 (red) in HT29 and HTOXAR3 cells. Nuclei were stained in blue. Objective lens: 40x immersion oil. Scale bar: 10 μm. Bar graphs represent mean ± SEM values, calculated from at least three independent experiments. *p-value < 0.05 and **p-value < 0.01; relative to protein expression in the corresponding sensitive cell line.

Previous results from our group suggested an important role for the NF-κB pathway in OXA resistance acquisition in *in vitro* models^12^. In the present work, we tried to demonstrate this hypothesis by first investigating the NF-κB basal status in 3 CRC Cell lines (HT29, LoVo and DLD1) and their corresponding OXA-resistant derived sub-lines (HTOXAR3, LoVOXAR3 and DLDOXAR3, respectively). As shown in Fig. 1 all the cell lines had constitutively phosphorylated p65 subunit at Ser536 which is considered an indicator of this pathway’s activity^9^. However, we observed an increase in this phosphorylation in HTOXAR3 (Fig. 1a,b) and DLDOXAR3 (Fig. 1a,d) as compared with their parental cell lines. Besides this, increased levels of phosphorylated IκBα at Ser32/36 residues, indicating degradation of this NF-κB inhibitor by the proteasome and p65 nuclear translocation, were also found in all three resistant cell lines in comparison with their respective parental ones. Taking into account that Survivin, Bcl-2 and Cyclin D1 have all been shown to be transcriptionally regulated by NF-κB^6^, we examined the basal expression levels of these proteins by western blot. We observed that all of them were significantly overexpressed in HTOXAR3 as compared to HT29 cells (Fig. 1a,b). In the case of the LoVo/LoVOXAR3 pair we observed a significant increase in Bcl-2 and Cyclin D1 protein expression in the resistant cell line (Fig. 1a,c) while only Survivin protein expression showed statistically significant differences between DLDOXAR3 and DLD1 (Fig. 1a,d). The Serine/threonine kinase Akt plays a critical role in proliferation and cell survival and its implication in the activation of anti-apoptotic mechanisms such as those driven by NF-κB is well documented^28^,^29^. In agreement with this, in our previous study we reported increased levels of *AKT1* mRNA in OXA-resistant cell lines^12^. Here we also demonstrate a significantly increased phosphorylation of Akt at Ser473 in HTOXAR3 as compared to HT29 cells (Fig. 1a,b) suggesting a possible role for Akt in the NF-κB pathway activation in the HTOXAR3 cell line.

Taken together, our results indicate that the NF-κB pathway is hyper-activated as a result of OXA resistance acquisition, especially in the HTOXAR3 resistant cell line. In addition, through immunocytochemistry experiments, we corroborated this finding since the HTOXAR3 cell line showed increased p65 nuclear staining compared to HT29 (Fig. 1e). Therefore, we used the HT29/HTOXAR3 pair as a model to perform most of the subsequent analysis.

### OXA induces NF-κB activation in CRC cells

**Figure 2:**
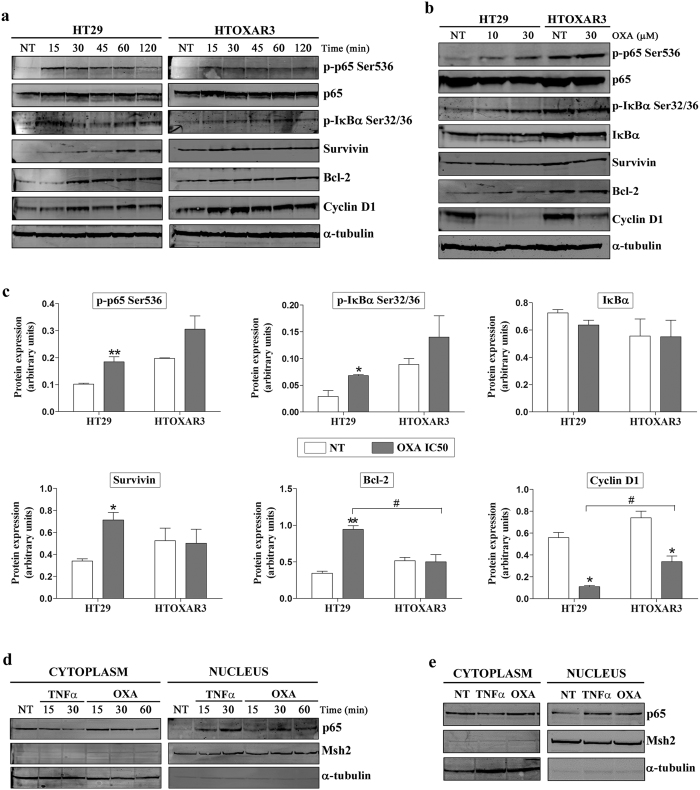
Effect of OXA treatment on NF-κB activation in HT29 and HTOXAR3 cells. Representative western blot images showing protein expression changes of phosphorylated p65 (p-p65 Ser536), phosphorylated IκBα (p-IκBα Ser32/36), Survivin, Bcl-2 and Cyclin D1 in HT29 and HTOXAR3 cells after treatment with 10 μM or 30 μM of OXA respectively, for 0 to 120 minutes (**a**) and for 24 hours **(b,c)** Bar graphs representing mean ± SEM expression changes of the indicated proteins after a 24-hour OXA treatment with the corresponding IC50 dose for HT29 (10 μM) and HTOXAR3 (30 μM). Alpha-tubulin was used as endogenous control. (**d**) Western blots showing p65 protein expression in cytoplasmic and nuclear extracts of HT29 cells after OXA treatment at short exposure times (0 to 60 min) and at 24 hours (**e**). TNFα was used as positive control at 50 ng/mL. Msh2 and α-tubulin were used as nuclear- and cytoplasmic-specific protein controls, respectively. NT: Non-treated cells. Results shown were obtained from at least 3 independent experiments. *p-value < 0.05 and **p-value < 0.01; as compared to NT. ^#^p-value < 0.05 relative to protein expression in the HT29 cell line.

NF-κB pathway could be activated through two mechanisms: signals that originate at cell receptors and signals that originate in the nucleus in response to DNA damage. Treatment with OXA potentiated NF-κB activation in different cell types, including CRC cell lines^10^,^30^. Accordingly, we assessed the phosphorylation status of p65 and IκBα, and the expression of three NF-κB target genes in HT29 and HTOXAR3 cell lines after short OXA-exposure times (0 to 120 min) and after 24 h of OXA treatment at 10 and 30 μM (IC50 values), respectively. We observed that OXA induced a fast phosphorylation of p65 and IκBα after the first 15 min of treatment and potentiated the expression of Bcl-2, Survivin and Cyclin D1 in both cell lines. Despite a slightly decrease in the expression of these proteins was observed after 30’ post-treatment, higher levels as compared to non-treated cells were maintained until at least 120’ after OXA treatment. Interestingly, we observed a trend to a more evident activation in HT29 as compared with HTOXAR3 cell line (Fig. 2a and Supplementary Fig. S1). In fact, these differences were more evident after a 24 h exposure since p65 and IκBα phosphorylation and Bcl-2 and Survivin expression increased in HT29 but not in HTOXAR3 cell line in a statistically significant way. In contrast, Cyclin D1 protein levels decreased significantly in both cell lines, this effect being more evident in the parental cell line. As previously described in HT29 cells^31^ we did not observe a significant IκBα protein degradation (Fig. 2b,c). When NF-κB is activated, the p65 subunit translocates to the cell nucleus. To confirm that OXA treatment promotes p65 nuclear translocation, HT29 cells were treated with OXA (10 μM) for varying lengths of times, and p65 expression in nuclear and cytoplasmic extracts was analysed by western blot. Nuclear p65 increased as early as 15 min after OXA exposure (Fig. 2d) and was sustained at least after 24 h of OXA treatment (Fig. 2e). TNFα was used as a positive control due to its ability to activate the NF-κB pathway.

Collectively, these results suggest that the combination of OXA with an NF-κB inhibitor could be an appropriate strategy to revert OXA resistance in our *in vitro* model. We tried that our work was the most closed to the clinics as possible. More than 700 different inhibitors of NF-κB have been reported, yet no blocker of this transcription factor has been approved for human use. We therefore selected the natural compound Curcumin (*diferuloylmethane*) in order to block NF-κB activation in our cells due to its strong ability to inhibit this pathway and its low toxicity profile in humans as reported in several phase I and II clinical trials^32^,^33^,^34^.

### Concomitant treatment OXA plus Curcumin for 24 hours reverts OXA resistance in CRC cells

**Figure 3:**
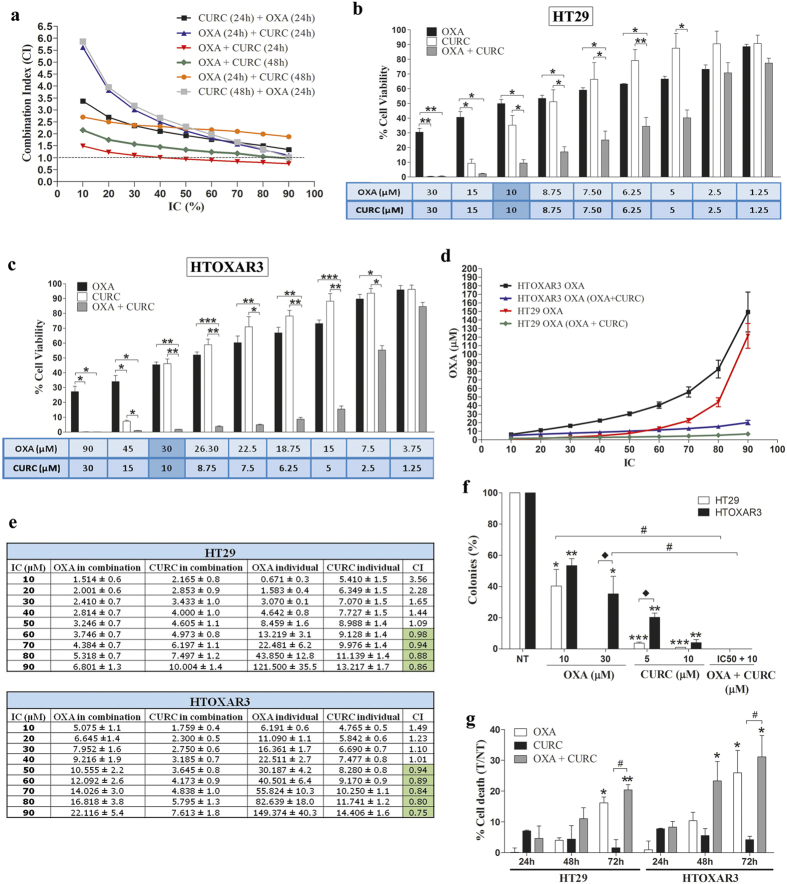
Combination of OXA and Curcumin in OXA-sensitive and –resistant CRC cell lines. (**a**) Graphic representation of the Combination Index (CI) values corresponding to different OXA plus Curcumin (CURC) treatment schedules in the HTOXAR3 cell line. (**b**) Bar graphs representing mean ± SEM percentage of cell viability after a 24-hour treatment with OXA, Curcumin or their concomitant combination at the indicated doses in HT29 and HTOXAR3 cells (**c**). *p-value < 0.05; **p-value < 0.01; ***p-value < 0.001 relative to the indicated treatment condition. (**d**) OXA doses (mean ± SEM) corresponding to indicated inhibitory concentrations (IC) in HT29 and HTOXAR3 cells as a single agent or when combined with Curcumin concomitantly for 24 h. (**e**) OXA and Curcumin doses (mean ± SD) corresponding to the indicated inhibitory concentrations (IC) when given as single agents or in a 24 h-concomitant schedule in HT29 and HTOXAR3. CI represents the combination index values in each case. Synergistic values (CI < 1) are highlighted in green. (**f**) Bar graph representing the percentage (mean ± SEM) of colonies in HT29 and HTOXAR3 cells after 24 h of the indicated treatments. *p-value < 0.05; **p-value < 0.01; ***p-value < 0.001 relative to NT (Non-treated cells). ^#^p-value < 0.05 relative to OXA individual treatment. ^*^p-value < 0.05 as compared to HT29. (**g**) Bar graph representing the percentage (mean ± SEM) of dead cells after 24, 48 and 72 h of treatment with OXA, Curcumin or their combination (as compared to non-treated controls) at their corresponding IC50 doses in HT29 and HTOXAR3 cell lines. *p-value < 0.05; **p-value < 0.01, relative to NT. ^#^p-value < 0,05, relative to Curcumin individual treatment.

A panel of 3 CRC cell lines and their respective OXA-resistant cells were treated with increasing concentrations of Curcumin for 24 h. Curcumin decreased cell proliferation of all cell lines in a dose-dependent manner (IC50 range 9.5–14.6 μM). Importantly, HTOXAR3 and LoVOXAR3 (Supplementary Fig. S2a,b) cells were more resistant to Curcumin treatment as compared with HT29 and LoVo cell lines whereas DLDOXAR3 cells were more sensitive than their parental sensitive cell line, DLD1 (Supplementary Fig. S2c). Next we wanted to determine the most synergistic combination between OXA and Curcumin. To do that, HTOXAR3 resistant cells were treated with different combination schedules, with the most synergistic being the concomitant treatment of both drugs for 24 h, where the CI was <1 from IC40 onwards, CI = 1 at IC30 and was slightly higher than 1 at very low doses (IC10 and 20) (Fig. 3a). Therefore, we decided to use this combination in order to evaluate the effect of Curcumin on the reversion of OXA-acquired resistance in our models. We observed that this schedule significantly decreased cell proliferation of both HT29 and HTOXAR3 cell lines (Fig. 3b,c) as compared with individual treatments. Remarkably, this reduction of cell viability was more evident in the HTOXAR3 cell line where in fact the combined treatment was able to revert the OXA-acquired resistance phenotype leading to a 65% reduction in the OXA IC50 (10.6 ± 2.2 μM with the combination vs. 30.2 ± 4.2 μM in individual treatment) and brought it closer to that of the HT29 sensitive cell line (8.45 ± 1.6 μM) (Fig. 3d). While in the HT29 cell line, the combination of Curcumin plus OXA was additive at the IC50 of these drugs and synergistic at doses higher than the IC50, in the resistant HTOXAR3 cells, the treatment started to be synergistic at the IC50 (Fig. 3e). Clonogenic assays revealed that while both drugs reduced the number of colonies as compared with untreated cells, their combination led to a complete absence of colonies in both HT29 and HTOXAR3 cells (Fig. 3f). In order to determine if these results could be explained by an increased cell death after the combined treatment, HT29 and HTOXAR3 cells were treated with OXA, Curcumin or their combination at their respective IC50 doses for 24, 48 and 72 h. As can be seen in Fig. 3g, OXA induced cell death rates below 25% after 72 h of treatment in both cell lines while in the case of Curcumin alone these rates were less than 10% in all cases. However the percentage of dead cells was increased after treatment with OXA plus Curcumin for 72 h in HT29 cells and for 48 and 72 h in HTOXAR3 only as compared with Curcumin alone. Similar results were obtained in the LoVo/LoVOXAR3 pair although an additive effect was observed only at high doses (IC90) of the two drugs in both cell lines (Supplementary Fig. S3).

In summary, these results suggest that the addition of Curcumin to OXA treatment sensitizes CRC cells to the latter, with this effect more evident in the OXA-resistant cell lines.

### Curcumin inhibits the OXA-induced activation of NF-κB and decreases the expression of NF-κB anti-apoptotic and pro-proliferative gene products

**Figure 4:**
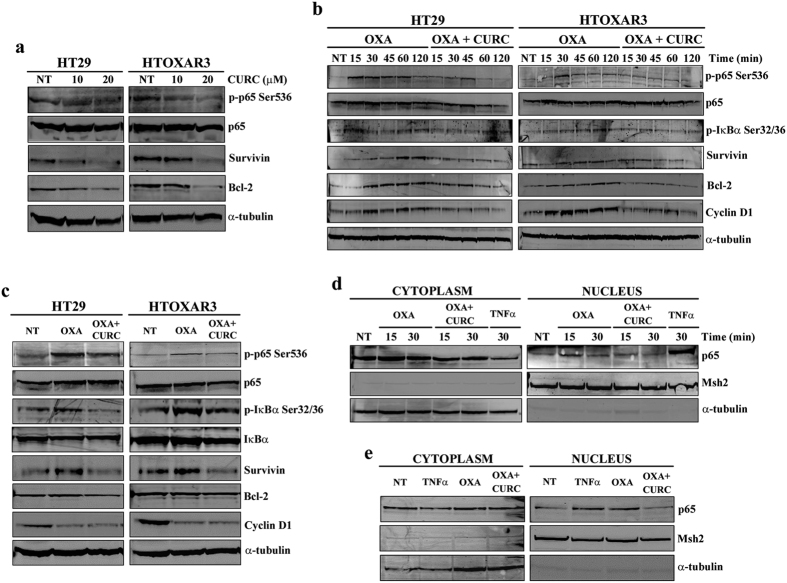
Effect of Curcumin on constitutive and OXA-induced NF-κB activation. (**a**) Western blot images of phosphorylated p65 (p-p65 Ser536), total p65, Survivin and Bcl-2 in HT29 and HTOXAR3 cells after a 24-hour treatment with 10 and 20 μM Curcumin (CURC). **(b)** Representative western blot images showing protein expression changes as indicated, in HT29 and HTOXAR3 cells after 24 h treatment with OXA or concomitant OXA plus Curcumin, for 0 to 120 minutes or 24 hours (**c,d**) Western blots showing p65 protein expression in cytoplasmic and nuclear extracts of HT29 cells after treatment with OXA (10 μM) or OXA + Curcumin (10 μM each) for short-time exposure (0 to 30 min) or 24 h (**e**). TNFα was used as positive control at 50 ng/ml. Msh2 and α-tubulin were used as nuclear- and cytoplasmic-specific protein controls, respectively. Images are representative of at least 3 independent experiments. NT: Non-treated cells.

In view of our results, we wanted to study the effect of the addition of Curcumin on OXA treatment at the molecular level. We first investigated the effect of Curcumin as a single agent on NF-κB inhibition. HT29 and HTOXAR3 cells were treated with Curcumin at 10 and 20 μM for 24 h. As expected, Curcumin decreased p65 phosphorylation and Survivin expression in a dose-dependent manner in both cell lines (Fig. 4a). Next, both cell lines were treated with OXA and OXA plus Curcumin at IC50 doses for short exposure times (0 to 120 min) and for 24 h. Results in Fig. 4b show that, in HT29/HTOXAR3 cells, the addition of Curcumin significantly decreased the OXA-induced phosphorylation of p65 (p < 0.001/p = 0.04) and IκBα (p < 0.05/p = 0.02) as well as the expression of Bcl-2 (p < 0.03/p < 0.01), Survivin (p < 0.001/p = 0.02) and Cyclin D1 (p < 0.001/p = 0.04) after short treatment times. Similar results were observed after a 24 h-treatment, although they did not reach statistical significance (Fig. 4c). We also studied how the addition of Curcumin affected the OXA-induced p65 nuclear translocation. As shown in Fig. 4d,e, levels of nuclear p65 were decreased in all cases when HT29 cells were treated with OXA plus Curcumin as compared with OXA alone.

These results suggest that at least in part, the effects of Curcumin alone and in combination with OXA may be mediated by the inhibition of the NF-κB signalling cascade.

### Treatment with Curcumin and OXA induces different gene expression patterns between OXA-sensitive and -resistant cell lines

Our results indicate that the combination of Curcumin and OXA is more effective and synergistic in HTOXAR3 cell line, in which the NF-κB pathway is hyper-activated, as compared with its sensitive-parental cell line, HT29. Given that NF-κB is a transcription factor, we wanted to investigate whether a specific transcriptomic pattern was associated with this differential effect. Total RNA from HT29 and HTOXAR3 cells was obtained after vehicle treatment and after a 24 h-treatment with OXA or OXA plus Curcumin at IC50 doses and global transcriptional profiling was assessed using microarrays. We were interested in genes that were altered as a consequence of the resistance acquisition process and at the same time, were differentially expressed after treatment with the combined treatment. At the significance cut-off thresholds (|FC| > 1.2 and q-value < 0.05) the results showed 4,237 regulated transcripts when comparing the two cell lines under basal conditions (among 36,204 genes including unique gene symbols and other transcripts). A total of 2,303 transcripts were up-regulated and 2,034 down-regulated in HTOXAR3 relative to HT29, confirming the involvement of multiple genes and pathways in OXA acquired resistance. There were 656 differentially regulated transcripts in the differential response analysis to combination treatment, 261 ‘up’ and 395 ‘down’ in HTOXAR3 relative to HT29 (Supplementary Table S2).

The intersection between both lists allowed us to identify 75 genes up-regulated by the combination treatment in HTOXAR3 relative to HT29 which were repressed under basal conditions, and 194 genes showing the opposite relationship (activated under basal conditions in HTOXAR3 cells and down-regulated by combination treatment in these cells than in the sensitive parental cell line, HT29) (Supplementary Figure S4). Exploratory functional enrichment analysis revealed the biological processes induced by the combination treatment (with GSEA overlap q-values < 0.00001): interferon gamma response, complement, epithelial to mesenchymal transition (EMT) and TNFα signalling via NF-κB in the 194 gene list and the TNFα signalling via NF-κB in the 75 gene list (Supplementary Table S3). This is in agreement with the central role of the NF-κB pathway in our OXA resistance acquisition model and supports the coherence of our dataset with prior evidence. In addition it would support the proposed mechanism of action by Curcumin acting through the same pathway.

**Figure 5:**
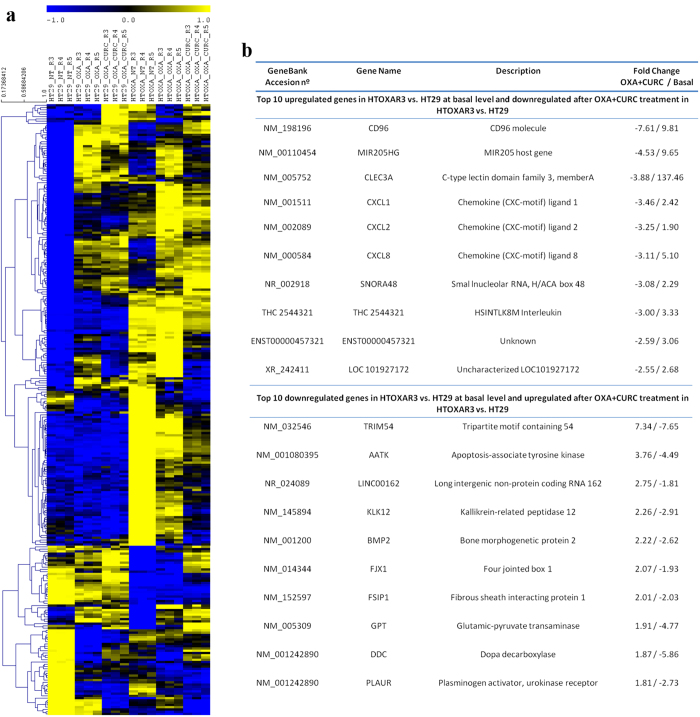
Gene expression patterns associated with OXA resistance and Curcumin + OXA synergism. (**a**) Hierarchical clustering of genes using pearson correlation as distance and average linkage with leaf order optimization. Cluster analysis was perfomed with TMEV version 4.8.1 using normalized expression values (mean subtracted log2 intensities divided by the standard deviation of the log2 intensities for each gene) (**b**) Table showing the top 10 up and down-regulated genes ranked by basal fold change between basal levels between HTOXAR3 and HT29 with regulation reversed by concomitant treatment.

**Figure 6:**
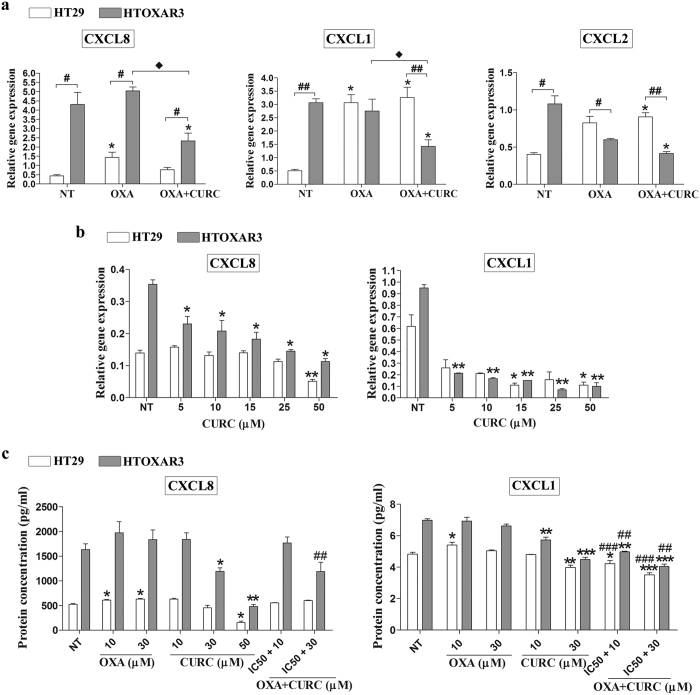
*CXCL1*, *CXCL8* and *CXCL2* gene expression and CXCL1 and CXCL8 secreted protein levels after treatment with OXA, Curcumin or both. (**a**) Bar graphs illustrating relative gene expression levels (mean ± SEM) of the indicated chemokines in non-treated (NT) and after OXA or OXA plus Curcumin (CURC) treatment at IC50 doses in HT29 and HTOXAR3 cells. Gene expression levels of β-actin were used as endogenous control. *p-value < 0.05 relative to NT, ^#^p-value < 0.05; ^##^p-value < 0.01, relative to HT29. ^**^ p-value < 0.05 relative to OXA. (**b**) Changes in chemokines’ gene expression after treatment with Curcumin at indicated doses. (**c**) Graph bars showing levels of secreted chemokines (mean ± SEM) measured by enzyme-linked immunosorbent assay (ELISA) in the supernatants of treated cells as indicated. *p-value < 0.05; **p-value < 0.01; ***p-value < 0.001 relative to NT. ^##^p-value < 0.01, ^###^p-value < 0,001 relative to OXA. Results were obtained from at least 3 independent experiments.

Among the top 10 genes fitting these criteria (Fig. 5) we observed that three pro-inflammatory CXC-chemokines, *CXCL8* (Interleukin-8), *CXCL1* (Gro-α) and *CXCL2* (Gro-β) that are primarily regulated by NF-κB^35^ were significantly up-regulated at basal level, and were more efficiently down-regulated after treatment with OXA plus Curcumin in the resistant cell line. Accordingly, functional enrichment analysis revealed cytokine signalling as one of the two molecular functions significantly overrepresented in this condition (Supplementary Figure S5). These results were validated through qRT-PCR experiments, confirming a basal overexpression of *CXCL8* (~10-fold), *CXCL1* (~6-fold) and *CXCL2* (~2.5-fold) in HTOXAR3 cell line as compared with HT29 (Fig. 6a). The three chemokines were up-regulated after OXA treatment only in HT29 cells (*CXCL8* 3-fold, *CXCL1* 6-fold and *CXCL2* 2-fold; Fig. 6a). In the case of *CXCL2*, we observed a down-regulation in expression after OXA treatment in HTOXAR3 cells. Interestingly, the OXA-induced expression of *CXCL8* and *CXCL1* was significantly attenuated by the addition of Curcumin in the resistant cell line but not in the sensitive one (Fig. 6a). Down-regulation of *CXCL2* with regards to basal conditions was also observed in the HTOXAR3 cell line after concomitant treatment (Fig. 6a). In view of these results, we investigated the effect of a 24 h-treatment with Curcumin as a single agent at different doses, on the expression of *CXCL8* and *CXCL1* observing a significant reduction in a dose-dependent manner only in the resistant cell line while in HT29 cells, this reduction was only observed at very high doses (Fig. 6b). ELISA-based analysis of HT29 and HTOXAR3 conditioned media revealed similar results at protein-secreted levels (Fig. 6c).

### *CXCL8* and *CXCL1* siRNA-mediated gene silencing sensitizes HTOXAR3 resistant cells through the inhibition of the NF-κB signalling pathway

**Figure 7:**
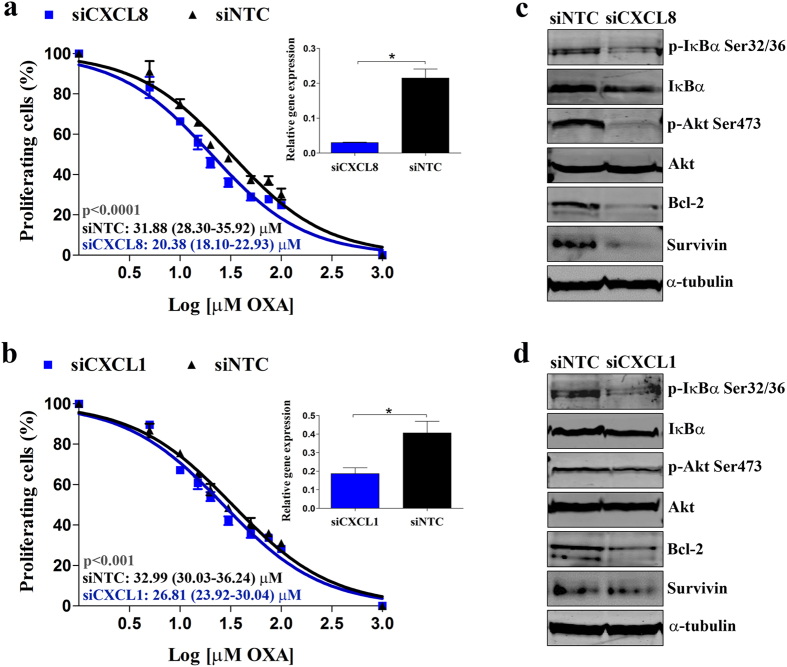
Effect of *CXCL8* and *CXCL1* siRNA-mediated gene silencing on OXA cytotoxicity and NF-kB signalling pathway activation in HTOXR3 cells. (**a**) Dose-response curves for HTOXAR3 cell line, after *CXCL8* and (**b**) *CXCL1* gene silencing, treated with 0-100 μM OXA for 24 hours (mean ± SEM). Insets show siRNA-mediated gene silencing efficiency at 48 h post-transfection. IC50 values are indicated as mean (95% CI). (**c**) Representative western blot images showing changes in phosphorylated IκBα (p-IκBα Ser32/36), phosphorylated Akt (p-Akt Ser473), Survivin, Bcl-2 and Cyclin D1 protein expression in HTOXAR3 cells under control (siNTC) and *CXCL8* (siCXCL8) or (**d**) *CXCL1* (siCXCL1) gene silencing. All results were obtained from at least 3 independent experiments.

To confirm the specific role of CXCL8 and CXCL1 on OXA resistance acquisition in HTOXAR3 cells, we transiently silenced their gene expression by using siRNA oligonucleotides. We assessed the cytotoxicity of OXA by MTT assay in control cells (siNTC) and *CXCL8* or *CXCL1* knockdown cells (siCXCL8 or siCXCL1, respectively). Forty-eight hours after gene silencing, cells were seeded in 96-well plates and treated for 24 h with doses of OXA ranging 0–100 μM. *CXCL8* and *CXCL1* knockdown efficiency was ~90% and ~60% at mRNA level, respectively (insets on Fig. 7a,b). Under these conditions, we found that *CXCL8* and *CXCL1* gene silencing in HTOXAR3 led to a 35% and a 20% decrease in OXA IC50 value as compared to siNTC cells, respectively (Fig. 7a,b).

CXCL8 and CXCL1 have been reported to act as autocrine growth factors through the induction of NF-κB transcriptional activity^10^,^36^,^37^,^38^. Thus, we investigated NF-κB activation by western blot under *CXCL8* and *CXCL1* gene silencing conditions. As expected, phosphorylated IκBα and Bcl-2 and Survivin protein expression levels were decreased in siCXCL8 and siCXCL1 cells as compared with siNTC cells. We also observed a decrease in Akt phosphorylation after silencing of both genes (Fig. 7c,d).

Globally, our results support first, the probable role of CXCL8 and CXCL1 chemokines in the OXA resistance acquisition process through the activation of the Akt/NF-κB pathway and second, their implication in the synergism and greater efficacy of OXA plus Curcumin treatment in OXA-resistant cells. Accordingly, the expression levels of *CXCL8* and *CXCL1* could be used as predictive markers of this combination’s effectiveness.

### Evaluation of CXCL8 and CXCL1 as predictive markers of OXA plus Curcumin treatment effectiveness

**Figure 8:**
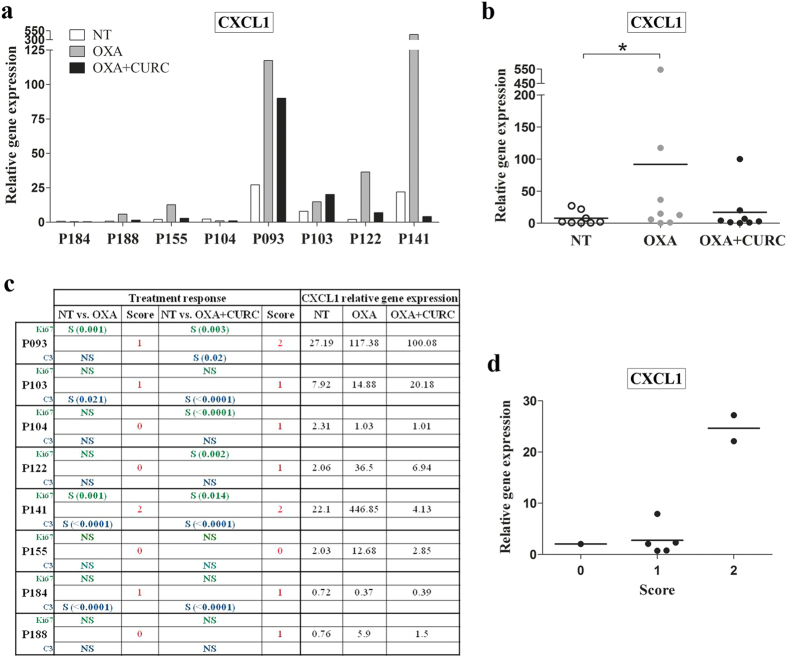
CXCL1 gene expression in explant cultures of patient-derived tissues after treatment with OXA or OXA + Curcumin. (**a**) Bar graph illustrating CXCL1 expression levels in 8 FFPE samples from explant cultures of CRC patients-derived liver metastases, that were treated with DMSO (NT), OXA or OXA plus Curcumin (CURC) for 24 h. (**b**) Scattergram reporting the expression levels of CXCL1 in NT, OXA or OXA plus Curcumin treated tissue explants. Horizontal lines represent mean values. (**c**) Table showing significative (S) or not significative (NS) changes in ki-67 (green) and cleaved caspase 3 (C3, blue) staining. Score 2 meant that ki-67 decreased and cleaved caspase increased significantly after the indicated treatment; score 1 meant that ki-67 decreased or cleaved caspase increased and score 0 meant that neither of them was changed. CXCL1 gene expression changes after NT, OXA or OXA + Curcumin treatment are also shown. (**d**) Scatter plot showing CXCL1 expression according to score values. Horizontal lines represent mean values. *p-value < 0.05 relative to NT (Wilcoxon test).

In order to evaluate the potential of CXCL8 and CXCL1 as predictive biomarkers of OXA plus Curcumin treatment response, we analysed gene expression of both chemokines in a panel of 8 FFPE samples from explant cultures of CRC patients-derived liver metastases, that were treated with OXA or OXA plus Curcumin or vehicle (DMSO) for 24 h. Response to each treatment was assessed by means of decreasing proliferation and/or apoptosis activation as indicated by ki-67 and cleaved caspase-3 inmunohistochemistry staining, respectively^34^. The RNA concentrations obtained from these samples ranged (20–75 ng/μL) and under our qRT-PCR conditions we were unable to detect *CXCL8* expression. According to our *in vitro* results, we observed an increase of CXCL1 expression in 6/8 explants treated with OXA that was attenuated by the addition of Curcumin in 5/6 explants (Fig. 8a,b). We then studied the expression patterns of *CXCL1* according to “response” to treatment. To do this, we assigned each case a score according to changes in ki-67 and cleaved caspase 3 staining after treatments (compared to vehicle). Thus, a score of 2 meant that ki-67 decreased and cleaved caspase 3 increased significantly after treatment; score 1 meant that ki-67 decreased or cleaved caspase 3 increased and score 0 meant that neither of them was changed according to this criteria. Interestingly, other authors have used a similar approach before^39^. As shown in Fig. 8c, explants p93 and p141 were the only ones with a score = 2 when treated with Curcumin + OXA and interestingly, they had the highest *CXCL1* basal levels as compared to explants with score 1 or 0. Although these results were obtained in a very few cases, they are in agreement with those obtained *in vitro* supporting the idea that those patients whose tumours present high *CXCL1* levels (basally or after treatment with OXA-based chemotherapy) would be suitable candidates to be treated with a combination of Curcumin plus OXA (Fig. 8d). Whether this also applies to CXCL8, remains to be elucidated. Further clinical studies are on going in order to investigate these hypotheses.

## Discussion

Chemotherapy schedules based on OXA combinations are essential in the clinical management of metastatic CRC patients. However, resistance phenomena remain one of the principal problems hindering treatment success. In previous work we found that the deregulation of the NF-κB signalling pathway could be an important mechanism of OXA-resistance acquisition in our *in vitro* models^12^. The role of elevated NF-κB activity in the resistance of CRC cells to OXA-induced cell death was previously demonstrated^30^ and in agreement with other studies^40^, results presented here indicate that NF-κB is constitutively active in all the CRC cell lines examined; however, we found that those cell lines with acquired resistance to OXA exhibited an increased activation of this pathway as compared to their matched sensitive parental cells, especially the HTOXAR3 resistant model, suggesting a role for NF-κB and its downstream targets in the development of the drug-resistant phenotype. This fact was reported to be associated with the acquisition of a mesenchymal phenotype along the OXA-resistance acquisition process^41^,^42^ and according to this, our exploratory functional enrichment analysis revealed EMT as one of the top hallmarks in the list of genes that were up-regulated in HTOXAR3 cell line in comparison with HT29 cells (Supplementary Table S3). Therefore our results could be explained in part by an EMT process in OXA resistant cells.

Similarly to other conventional anticancer drugs^43^,^44^, administration of OXA further potentiates NF-κB activity, increasing transcriptional regulation and expression of anti-apoptotic genes^10^. Consistent with this, our results indicate that OXA activates NF-κB in HT29 and HTOXAR3 cell lines through the classical pathway: IκBα and p65 phosphorylation, p65 nuclear translocation and expression of NF-κB-regulated genes, including Bcl-2 and Survivin. However, in agreement with other authors, we did not observe IκBα degradation after OXA exposure. It seems that HT29 cells display a NF-κB-dependent inflammatory response with final induction of target genes expression but at the same time, they are unable to degrade IκBα, probably because of a defective proteasome system or by low levels of IKK activation^31^,^45^,^46^. Importantly, we observed that NF-κB activation occurred very rapidly after OXA treatment, probably through the activation of death receptors, such as TNFR family^47^, but it was significantly sustained after treatment for 24 h only in the parental-sensitive HT29 cell line. This activation after OXA treatment could be due to the well-known capacity of OXA to induce both oxidative cellular stress and DNA damage. Differences observed between both cell lines could be explained by the tolerability of HTOXAR3 to this damage; this would be reinforced by the fact that most of the DNA repair genes found to be differentially expressed in our microarray experiments between sensitive and resistant cells, were overexpressed in the latter (17/25) while expression of genes encoding for DNA damage response proteins, were down-regulated in this cell line indicating an adaptation to DNA damage and in consequence contributing to an attenuated NF-κB activation after OXA treatment (Supplementary Table S4). We also observed a drastic reduction in Cyclin D1 protein levels after OXA treatment. Cyclin D1 regulates the G1 to S phase transition, being synthesized during the G1 phase and degraded as the cell enters the S phase^48^. As other authors and we have reported, a 24-h OXA treatment causes S-phase accumulation in HT29 cells^49^,^50^; this effect could explain the Cyclin D1 protein decrease after this time of OXA treatment. Targeting of NF-κB has been explored extensively as a therapeutic strategy against cancer and Curcumin has been demonstrated to suppress NF-κB activation and NF-κB-dependent gene expression^6^. As expected, we found that Curcumin suppressed constitutive and OXA-induced NF-κB activation as well as proliferation^13^,^21^ and it was able to sensitize CRC cells to OXA treatment. Indeed, the concomitant treatment of Curcumin and OXA led to a significant suppression of cell proliferation and colony formation as compared to either treatment alone in LoVo and HT29 cells. The combination was especially efficacious in their corresponding OXA-resistant derived LoVOXAR3 and HTOXAR3 cells. This observation was also reported by Howells *et al.*^19^. Drug combination experiments revealed that the concomitant treatment was more synergistic in the HTOXAR3 cell line than in the HT29 cell line and in fact, this treatment reverted the resistance to OXA in the former. However, under the same conditions, no synergism was found between the two drugs in the LoVo/LoVOXAR3 pair. These differences could be partly explained by differences of genetic features between the two *in vitro* models, by the fact that the NF-κB pathway was not so evidently hyperactivated in LoVOXAR3 cells or by the necessity to test specific combination schedules in these cell lines in order to find the most synergistic one in this model. We found this effect was not associated with an increase in cell death after 24 h treatment which was in line with other studies^50^. Taken together, these results are of paramount interest because they suggest that a suboptimal concentration of OXA could be used in combination with Curcumin leading to similar efficacy but minimizing toxicity.

We then wanted to know whether a specific gene expression pattern in the HTOXAR3 cell line was associated with the greater efficacy of OXA plus Curcumin. Three NF-κB-transcriptionally regulated CXC-chemokines, *CXCL8*, *CXCL1* and *CXCL2*, were highly overexpressed as a result of OXA-resistance acquisition in the HTOXAR3 cell line and at the same time were specifically down-regulated after treatment with OXA plus Curcumin in these cells. These pro-inflammatory chemokines play an important role in regulating CRC progression, angiogenesis and metastasis^51^, processes that have been associated with OXA resistance^42^. The effect of chemotherapy on *CXCL8*^52^,^53^, *CXCL1* and *CXCL2*^54^ gene transcription has been described elsewhere. For example, Wilson *et al.* showed that OXA treatment induced *CXCL8* and *CXCL1* gene expression and potentiated NF-κB activation^10^. Similarly, we show that OXA treatment strongly induced CXCL8 and CXCL1 gene and protein expression only in the HT29 OXA-sensitive cells, which was in agreement with results of NF-κB activation after 24 h of treatment with OXA only in this cell line. Curcumin has been demonstrated to down-regulate the expression of CXCL1 and CXCL8^55^,^56^, interestingly the addition of Curcumin to OXA treatment suppressed these chemokines’ expression only in the OXA-resistant HTOXAR3 cell line. In summary, this finding suggests the implication of these two chemokines in the observed greater efficacy and synergism of OXA and Curcumin in the OXA-resistant cells. We further demonstrated that overexpression of *CXCL8* and *CXCL1* is indeed affecting OXA-resistance acquisition since their gene silencing led to a significant although slight sensitization of HTOXAR3 cells.

CXCL8 and CXCL1 can be secreted and promote positive autocrine and/or paracrine feedback loops through their binding to the CXC receptors CXCR1 and CXCR2 which in turn activates the NF-κB signalling pathway^10^. We show that this also happened in our model as CXCL8 and CXCL1 gene silencing was associated with decreased phosphorylation of IκBα and reduced protein levels of Bcl-2 and Survivin in HTOXAR3 cells. Under these conditions we also observed a decrease in Akt phosphorylation, which suggests that at least in part, the observed NF-κB hyperactivation in the resistant cell line could be a result of an acquired increased activation of Akt. These results are supported by prior studies indicating that overexpression of *CXCL8* causes phosphorylation of Akt and NF-κB activation in the HCT116 CRC cell line^57^. This signalling pathway has been associated with resistance to OXA in prostate cancer cells^10^ and treatment with a CXCR2 antagonist such as SCH-527123, sensitizes cells to OXA in preclinical colon cancer models^58^. We propose that not only the inhibition of NF-κB *per se*, but also the binding of chemokines to receptors that activate this pathway could be useful strategies in increasing the efficacy of OXA in CRC. Finally, we tried to translate our results to the clinical setting. Facing the impossibility of finding samples from patients treated with Curcumin, we took advantage of previous work from our collaborators in which they utilised patient-derived colorectal liver metastases explant cultures treated with different schedules containing Curcumin^34^. In order to mimic our *in vitro* experiments, we chose those explants that were treated with OXA or OXA + Curcumin. We found that, treatment with OXA also induced the expression of CXCL1 that was repressed by the addition of Curcumin. Interestingly, the explants with best “response” to OXA + Curcumin, were those with the highest basal levels of CXCL1, suggesting that this chemokine could be a good predictive marker for this treatment. These results are not definitive yet they lead us to hypothesize that i) in some cases, the addition of Curcumin to OXA could lead to a more efficacious treatment, permitting dose reduction of the latter and avoiding toxicity; ii) combinations of Curcumin + OXA could be an effective treatment in metastatic CRC patients, including those that had progressed to an OXA-based first line. In this case, we have to be careful and take into account that those patients developing toxicity to the first-line treatment would not be candidates to a second-line with OXA and iii) the measurement of CXCL1 (and probably CXCL8) in tumours (but also in serum) could serve to identify those patients that are more likely to respond to OXA + Curcumin. Clinical trials such as the one that is being coordinated by the Howells’ group in Leicester (NCT01490996; www.ClinicalTrials.gov) are warranted in order to validate these results and to establish the best conditions to include this compound in the clinical management of CRC patients.

## Materials and Methods

### Cell lines

Human tumor-derived colorectal adenocarcinoma HT29, LoVo, and DLD1 cell lines (American Type Culture Collection) were used as the parental cells to obtain the oxaliplatin-resistant sublines HTOXAR3, LoVOXAR3 and DLDOXAR3, respectively as a result of continuous exposure to increasing doses of OXA as described previously^12^. Cell lines were grown as monolayer in RPMI 1640 (Invitrogen, Life Technologies) medium (DLD1 and DLDOXAR3) and DMEM (Invitrogen, Life Technologies) medium (HT29 and HTOXAR3) supplemented with 10% of heat-inactivated FCS (Reactiva), 400 units/mL penicillin, 40 μg/mL gentamicin, 2 mmol/L L-glutamine (Sigma), and 10 mmol/L HEPES (Sigma). LoVo and LoVOXAR3 cells were grown in Ham’s F-12 (Invitrogen, Life Technologies) with 20% FCS (Reactiva), 200 units/mL penicillin, and 20 μg/mL gentamicin.All cell lines were cultured at 37 °C in a humidified atmosphere of 5% CO_2_. Cells were periodically tested for Mycoplasma contamination and were authenticated by short tandem repeat profiling.

### Samples from explant cultures of patient-derived tissues

FFPE explant cultures were a gift of Dr. Lynne Howells (University of Leicester). Procedures for their obtaining, culture, treatment and response evaluation are described in^34^. These samples were obtained from tumours from patients undergoing surgical resection for colorectal liver metastases at University Hospitals of Leicester NHS Trust. Ethical approval for this fully anonymised, excess tissue study was granted by Leicestershire, Northamptonshire and Rutland Ethics Committee (REC reference 09/H0402/45). Additionally, the Clinical Research Ethical Committee from Hospital Germans Trias i Pujol provided approval for the study.

### Drugs

OXA and Curcumin (Sigma) were prepared in water and in absolute ethanol respectively at a concentration of 10 mM and stored at −20 °C. Further dilutions of each drug were made in culture medium to final concentrations before use.

### Western blot analysis

Cells were homogenized in RIPA plus buffer [Phosphate Buffered Saline (PBS); NP-40 1%; Na deoxycholate 0.5%; SDS 0.1%; EDTA 1 mM; NaF 50 mM; NaVO_3_ 5 mM] containing a cocktail of EDTA-free protease inhibitors (Roche). Protein concentration was determined by the Bradford method by using the DC™Protein Assay (Bio-Rad) and bovine serum albumin as a standard. Fifty micrograms of protein were loaded and subjected to electrophoresis in 10% SDS-PAGE gels (Invitrogen, Life Technologies) and transferred onto PVDF membranes (Bio-Rad). After 1 h of blocking (LICOR Biosciences) membranes were incubated overnight at 4 °C using specific primary antibodies against phospho-p65 Ser536 (Merck-Millipore, 1:2000), p65 (Cell Signaling, 1:1000), phospho-IκBα Ser32/36 (Cell Signaling 1:1000), IκBα (Abcam, 1:1000) Bcl-2 (Abcam, 1:1000), Cyclin D1 (Abcam, 1:1000), Survivin (Abcam, 1:1000), phospho-Akt (Cell Signaling, 1:1000) and Akt (Cell Signaling, 1:1000). Mouse monoclonal anti-α-tubulin antibody (Sigma Aldrich, 1:20000) was used as internal and cytoplasmatic control. Anti-Msh2 antibody (Calbiochem, 1:500) was used as nuclear protein control. Membranes were incubated with IRDye rabbit and mouse secondary antibodies (1:10000) (LICOR Biosciences) for 50 minutes protected from the light. Membranes were scanned by using an Odyssey Imaging System and analyzed with Odyssey v2.0 software (LICOR Biosciences).

### Preparation of nuclear extracts

The nuclear extracts were prepared according to Schreiber *et al.*^59^. Briefly, cells were washed with cold PBS and suspended in 0.4 ml hypotonic lysis buffer containing protease inhibitors and 0.5% Nonidet P-40 for 30 minutes. The homogenate was centrifuged, and supernatant containing the cytoplasmic extracts was stored frozen at −80 °C. The nuclear pellet was washed twice with lysis buffer and then resuspended in 50 μl ice-cold nuclear extraction buffer. After 30 minutes of intermittent mixing, the extract was centrifuged, and supernatants containing nuclear extracts were secured. The protein content was measured by the Bradford method by using the DC™Protein Assay (Bio-Rad).

### Cell viability Assay

The cytotoxicity of OXA, Curcumin and combination of both was assessed by the 3-(4, 5-dimethylthiazol-2-yl) 2,5-diphenyltetrazolium bromide (MTT) test (Roche Diagnostics). Cells were seeded in 96-well microtiter plates (Nunc) at a density of 1,500 (HT29 and HTOXAR3), 3,000 cells/well (LoVo and LoVOXAR3) and 4,000 cells/well (DLD1 and DLDOXAR3). Cells were treated with different drug concentrations for 24 h. Subsequently, the drug was removed and fresh media was added to each well. Cells were allowed to grow for 72 h. MTT was then added and doses for each fraction of survival (ranging from 10% to 90% of cell viability) were determined in each cell line by the median-effect line method. The maximum concentration of absolute ethanol (Curcumin vehicle) used was 0,3% which did not affect cell proliferation in our experiments.

### Analysis of combined drug effects

Cell viability was assessed by MTT assay, as described above, and data were analyzed by Chou and Talalay method^60^. The analysis of combined drug effects was performed in each experiment with serial dilution of both drugs administered at doses that typically correspond to 1/8, 1/4, 1/2, 5/8, 3/4, 7/8, 1, 1.5 and 3 of the individual IC50 values. Fractional survival was then calculated by dividing the number of cells in drug-treated plates by the number of cells in control plates. Data was subsequently analyzed by the method of Chou and Talalay. For each level of cytotoxicity, a parameter called combination index (CI) was then calculated according to the following equation: Ci*f* = D1/(Df)1 + D2/(Df)2 + α·D1·D2/(Df)1·(Df)2; where (D1) and (D2) are the concentrations of the combination required to produce survival *f*; (Df)1 and (Df)2 are the concentrations of the individual drugs required to produce f, and α = 1 or 0 depending on whether the drugs are assumed to be mutually nonexclusive or mutually exclusive, respectively, in their action. For Curcumin and OXA combination we considered an α = 1 since they have a mutually exclusive mechanism of action. According tot this method, synergism is indicated by CI < 1, antagonism by a CI > 1, and additivity by CI = 1.

### Colony-formation assay

Colony-formation assays were also performed to evaluate the cytotoxicity of OXA, Curcumin and their combination. Briefly, cells were plated in 6-well plates at a density of 300 cells/well (HT29 and HTOXAR3) or 500 cells/well (LoVo and LoVOXAR3) and allowed to attach. The next day, different dilutions of the corresponding drugs were added for 24 h. After exposure, cells were washed in PBS and cultured in free media until colonies were formed (10 days). Cells were subsequently washed, fixed with a Methanol/acetic acid (3:1) solution for 10 min and stained with a solution of crystal violet (0,5%) for 10 min. After staining, cells were thoroughly washed with PBS. Colonies were counted manually.

### Gene silencing

Interfering RNA (siRNA) was used to generate specific knockdowns of *CXCL8* and *CXCL1* in the HTOXAR3 cell line. Cells were seeded at 60% confluence in serum and antibiotic-free OptiMem medium (Invitrogen, Life Technologies) in 6-well plates. *CXCL8* and *CXCL1* were transiently silenced by using a pool of 4 different siRNAs (Smartpool On-target plus: *CXCL8* siRNA, #L-004756; *CXCL1* siRNA, #L-003898 ;Dhamacon, GE, respectively). As a transfection agent we used Lipofectamine RNAiMAX (Invitrogen, Life Technologies) according to the manufacturer’s instructions. A silencer negative transcription control (Cat No. AM4611; Ambion) was introduced in each experiment. Following 24 h of transfection, medium was replaced with full DMEM medium supplemented with serum and antibiotic. Validation of *CXCL8* and *CXCL1* knockdown was assessed by qRT-PCR. In fine-tuning experiments, non-transfected cells (Mock) were introduced to ensure that transfection had minimal effects on gene expression, proliferation and cell viability.

### Quantitative RT-PCR

Gene expression experiments were performed as described in previous work^12^. Retrotranscription was performed with MMLV reverse transcriptase (Invitrogen, Life Technologies) in accordance to the manufacturer’s instructions. Then, 2 μL of each RT reaction was amplified in a LightCycler^®^ 480 PCR system (Roche), using the KAPA SYBR Fast Master Mix (KAPA Biosystems, Woburn, MA, USA). Primer pairs used are listed in Supplementary Table S1. The PCR product size generated with these primers was 155 bp for CXCL8, 104 bp for CXCL1 and 113 bp for CXCL2. Relative gene expression quantification was calculated according to the comparative Ct method as described elsewhere using β-Actin (Applied Biosystems) as endogenous control. In all experiments, only triplicates with a Ct value lower than 0.20 SD were accepted. In addition, for each sample analyzed, a retrotranscriptase minus control was run in the same plate to ensure absence of genomic DNA contamination.

### ELISA assay

Culture supernatants were collected 24 h post-treatment with OXA, Curcumin or their combination. *CXCL8* and *CXCL1* secretion was measured with the IL-8 OptEIA ELISA Set (Becton Dickinson) and the RayBio Human GRO-alpha ELISA kit (RayBiotech), respectively; following the manufacturer’s instructions.

### Propidium iodide test

OXA and Curcumin-induced cell death was measured by using Propidium Iodide (PI). After 24, 48 and 72 h of treatment with OXA, Curcumin or the combination of both, cells were harvested with Accutase (ref. A11105-01, Invitrogen) and resuspended in cold PBS with a PI concentration of 3 μM. The PI fluorescence was determined on a FACSCanto II flow cytometer (Becton Dickinson Immunocytometry System).

### Immunofluorescence analysis

Subcellular localization of p65 (NF-κB) was detected by immunofluorescence, as described in previous work^49^.

### Microarray gene expression profiling

We used Agilent HumanV2 Microarrays for global gene expression profiling. 100 ng of total RNA was used as input for reverse transcription. cRNA was then fluorescently tagged with Cy3-CTP nucleotide by *in vitro* transcription using the low input quick Amp Labeling kit followed by hybridization, washing, scanning and image extraction using commercial software; all steps were performed strictly following the manufacturer’s instructions (Agilent, Palo Alto, CA). Subsequent data pre-processing and analysis was as follows: raw data intensities were background corrected using normexp, and log2intensity distributions were made comparable between samples using quantile normalization. Differential gene expression was assessed by fitting to an empirical Bayesian linear model. Statistical significance in differential gene expression was determined by setting a false discovery rate applying the Benjamini and Yekutiely method for multiple testing adjustment correction. For analysis we used the Limma package and the R statistical programming environment^61^.

We analyzed total RNA obtained from 3 biological replicates of 3 conditions: basal without treatment, OXA individual treatment and OXA plus Curcumin in 2 cell lines (HT29 and HTOXAR3). Data are publicly available and can be accessed with the GSE76092 identifier in the GEO database (http://www.ncbi.nlm.nih.gov/geo/).

To look for differentially expressed genes following desired patterns of gene expression we first applied a dual filter applying as cut-off an absolute fold change |FC| > 1.2 and a q-value < 0.05 on each specific comparison. Then the intersection between lists was computed and Venn diagrams where plotted using Venny 2.02 (http://bioinfogp.cnb.csic.es/tools/venny/index.html)

### Functional enrichment analysis

To explore relevant enrichment in gene ontology categories within the genes found in the intersection between significantly regulated gene list, we used GOrilla web server tool^62^,^63^. For independent confirmation we also used the GSEA^64^ gene set investigation tool (http://software.broadinstitute.org/gsea/msigdb/annotate.jsp) and computed significant overlaps with GO ‘C5’ molecular function ‘MF’ gene sets as well as the ‘H’ gene set collection (hallmarks, a collection of 50 sets representing specific well defined biological states or processes identified by extracting coherent representatives from gene set overlaps in the version available November 20 2015 http://www.broadinstitute.org/cancer/software/gsea/wiki/index.php/MSigDB_v5.0_Release_Notes)

### Statistical analysis

Data are presented as mean ± SEM of at least 3 independent experiments and the statistical analysis was performed with Graphpad Prism V.4 software. Statistical differences between IC50 were determined by graphic representation of dose-response curves and subsequent non-linear regression analysis and F-test. Comparisons among different experimental conditions were carried out through the T-student or two-way ANOVA test followed by Bonferroni post-test. Values of p ≤ 0.05 were considered significant.

## Additional Information

**How to cite this article**: Ruiz de Porras, V. *et al.* Curcumin mediates oxaliplatin-acquired resistance reversion in colorectal cancer cell lines through modulation of CXC-Chemokine/NF-κB signaling pathway. *Sci. Rep.* **6**, 24675; doi: 10.1038/srep24675 (2016).

## Supplementary Material

Supplementary Information
