# Supplementary material for: Curcumin mediates oxaliplatin-acquired resistance reversion in colorectal cancer cell lines through modulation of CXC-Chemokine/NF-κB signalling pathway
Source: Sci Rep. 2016 Apr 19;6:24675. doi: 10.1038/srep24675 (PMC4835769; doi:10.1038/srep24675)
Supplement: Supplementary Information [file srep24675-s1.pdf]

Curcumin mediates oxaliplatin-acquired resistance reversion in colorectal cancer cell lines through modulation of CXC-Chemokine/NF-κB signalling pathway.

Vicenç Ruiz de Porras[1], Sara Bystrup[1], Anna Martínez-Cardús[2], Raquel Pluvinet[3], Lauro Sumoy[3], Lynne Howells[7], Mark I. James[7], Chinenye Iwuji[7], José Luis Manzano[1,4], Laura Layos, Cristina Bugés, Albert Abad[6] and Eva Martínez-Balibrea[1,5*]

1. Translational research in digestive tumors group. Laboratory of Molecular Cancer Biology, Health Sciences Research Institute of the Germans Trias i Pujol Foundation (IGTP), Can Ruti Campus, Ctra. Can Ruti- Camí de les escoles s/n, 08916, Badalona, Spain

2. Cancer Epigenetics and Biology Program (PEBC), Bellvitge Biomedical Research Institute (IDIBELL), Gran Via de l'Hospitalet, 199. 08908 L'Hospitalet de Llobregat, Barcelona, Spain

3. Genomics and Bioinformatics Unit, Institute for Predictive and Personalized Medicine of Cancer (IMPPC), Can Ruti Campus, Ctra. Can Ruti- Camí de les escoles s/n, 08916, Badalona, Spain

4. Medical Oncology Service, Catalan Institute of Oncology (ICO) University Hospital Germans TriasiPujol, Ctra. Can Ruti- Camí de les escoles s/n, 08916, Badalona, Spain.

5. Program Against Cancer Resistance (ProCURE), Catalan Institute of Oncology.

6. Oncology Unit, Hospital CIMA Sanitas, Barcelona, Catalonia, Spain.

7. Dept Cancer Studies, University of Leicester, Leicester Royal Infirmary, Lancaster Road, LE1 9HN, Leicester, UK.

Supplementary Figure S1

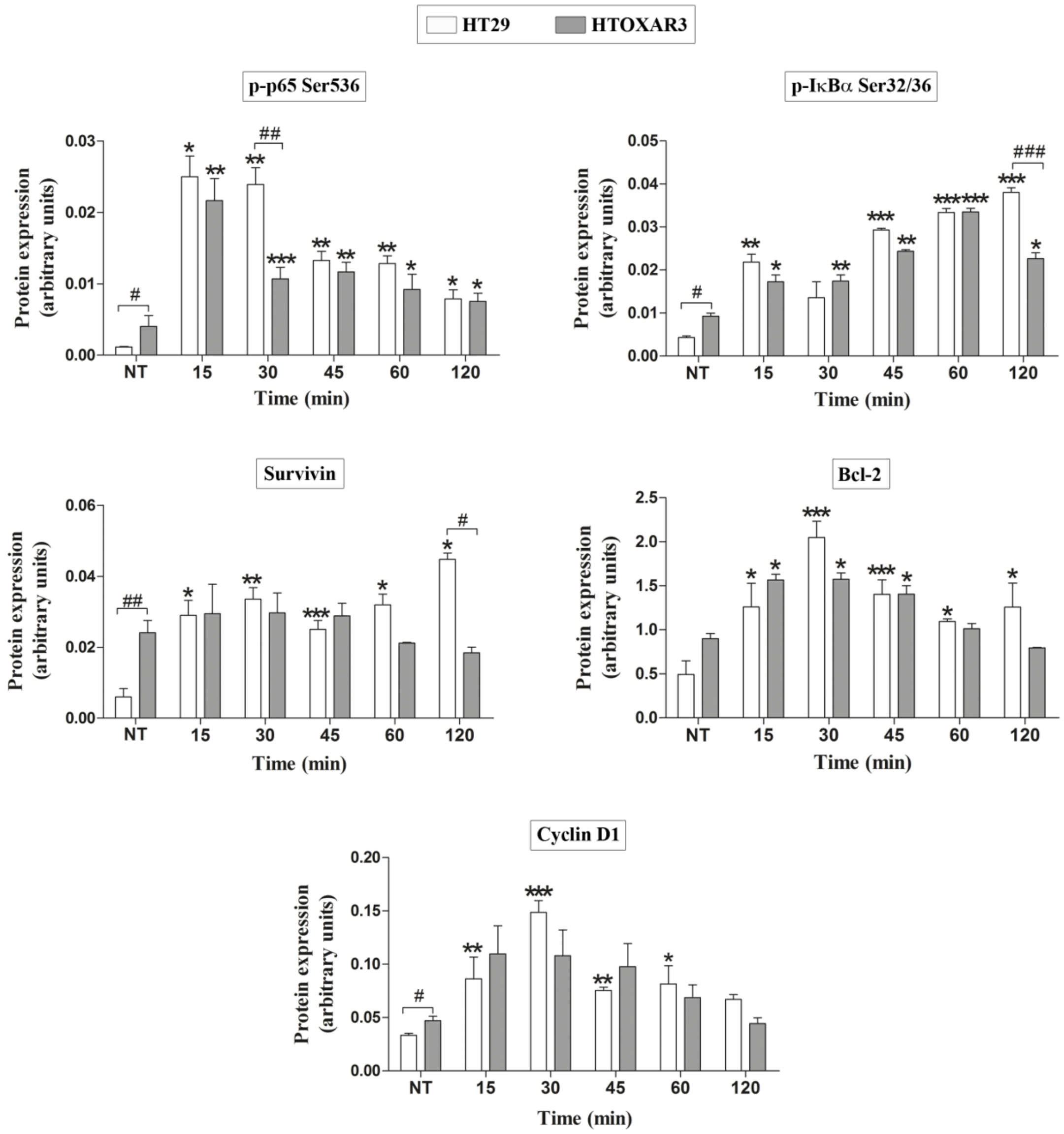

Supplementary Fig. S1. Effect of OXA treatment on NF-κB activation in HT29 and HTOXAR3 cells. Bar graphs showing levels (mean ± SEM) of phosphorylated p65 (p-p65 Ser536) phosphorylated IκBα (p-IκBα Ser32/36), Survivin, Bcl-2 and Cyclin D1 in HT29 and HTOXAR3 cells after 10 or 30 μM OXA treatment, respectively, for 0 to 120 min. Alpha-tubulin was used as endogenous control. Values are the result of at least three independent experiments. *p-value < 0.05; **p-value < 0.01; ***p-value < 0.001 relative to NT (Non-treated cells). #p-value < 0.05, ##p-value < 0.01 relative to corresponding protein expression in HT29 cell line.

Supplementary Figure S2

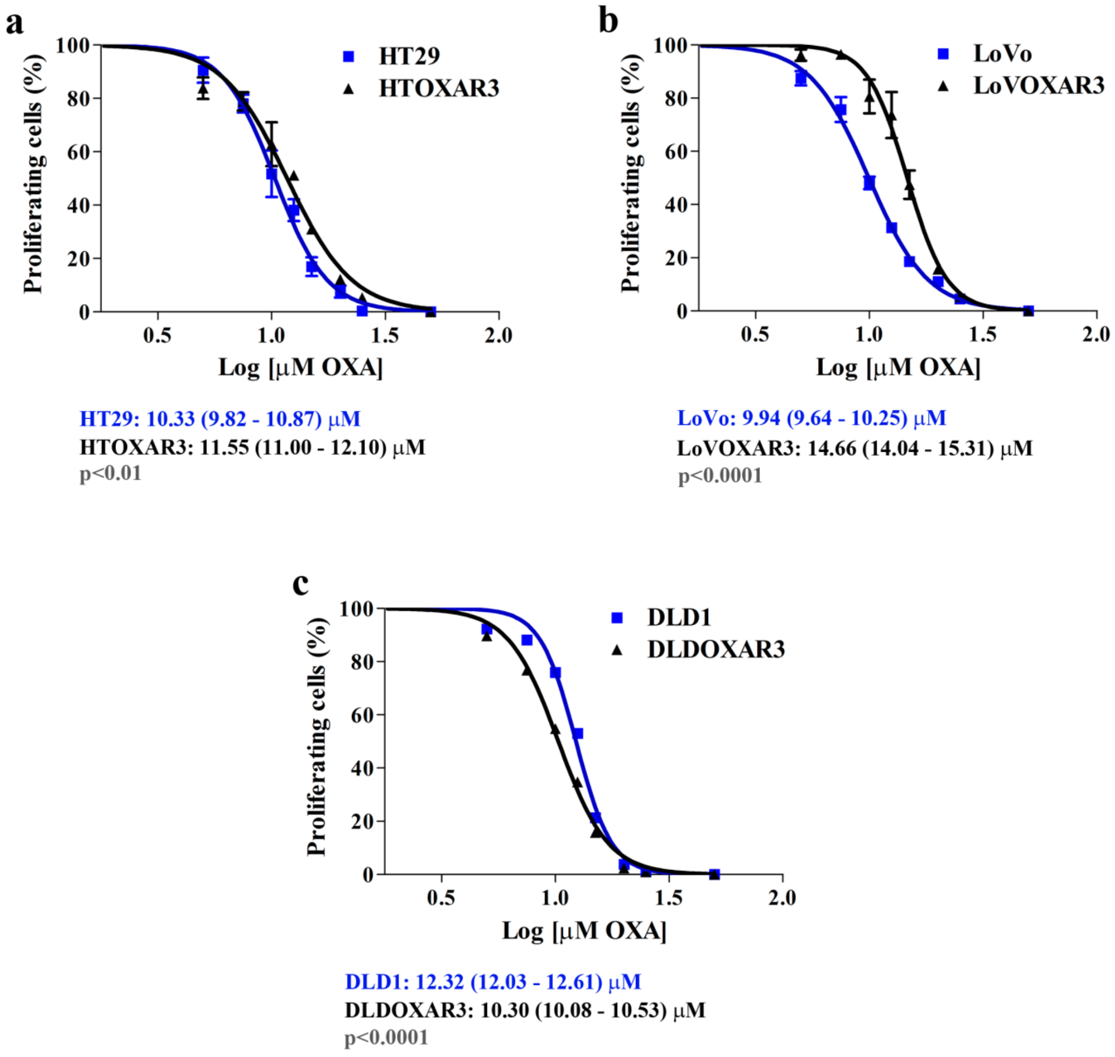

Supplementary Fig. S2. Effect of Curcumin treatment on CRC cells proliferation. **(a)** Dose response curves for HT29/HTOXAR3, **(b)** LoVo/LoVOXAR3 and **(c)** DLD1/DLDOXAR3 after Curcumin treatment at 0-50 μM for 24 h (mean ± SEM). IC50 values are indicated as mean (95% CI). All results were obtained from at least 3 independent experiments.

Supplementary Figure S3

a

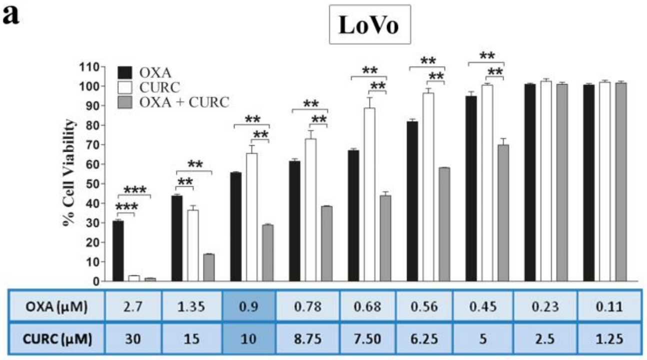

b

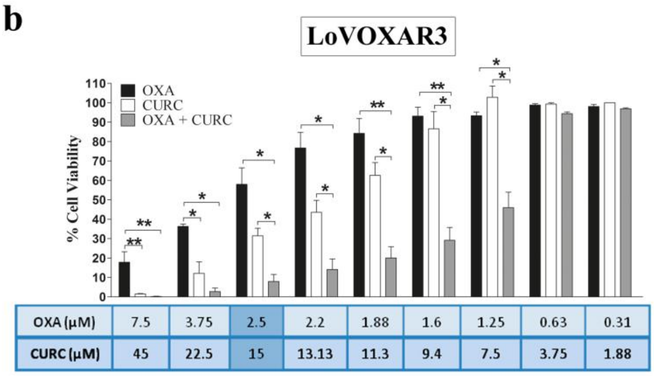

c

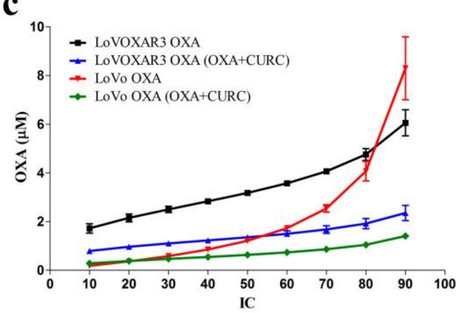

d

LoVo					
IC (μM)	OXA in combination	CURC in combination	OXA individual	CURC individual	CI
**10**	0.285 ± 0.0	3.178 ± 0.1	0.182 ± 0.0	7.912 ± 1.5	2.34
**20**	0.383 ± 0.0	4.275 ± 0.2	0.365 ± 0.1	9.478 ± 1.6	1.86
**30**	0.466 ± 0.0	5.196 ± 0.3	0.582 ± 0.1	10.688 ± 1.6	1.63
**40**	0.546 ± 0.0	6.098 ± 0.3	0.853 ± 0.0	11.794 ± 1.7	1.49
**50**	0.633 ± 0.0	7.062 ± 0.4	1.214 ± 0.0	12.911 ± 1.7	1.38
**60**	0.733 ± 0.0	8.178 ± 0.4	1.727 ± 0.1	14.134 ± 1.7	1.29
**70**	0.860 ± 0.0	9.599 ± 0.5	2.539 ± 0.2	15.600 ± 1.7	1.21
**80**	1.046 ± 0.1	11.669 ± 0.6	4.069 ± 0.6	17.597 ± 1.7	1.15
**90**	1.403 ± 0.1	15.653 ± 0.8	8.294 ± 1.8	21.098 ± 1.7	1.09

LoVOXAR3					
IC (μM)	OXA in combination	CURC in combination	OXA individual	CURC individual	CI
**10**	0.793 ± 0.1	4.722 ± 0.4	1.721 ± 0.5	6.375 ± 1.6	1.88
**20**	0.965 ± 0.1	5.753 ± 0.5	2.151 ± 0.4	7.841 ± 2.1	1.79
**30**	1.101 ± 0.1	6.566 ± 0.6	2.501 ± 0.3	9.001 ± 2.6	2.06
**40**	1.227 ± 0.1	7.322 ± 0.8	2.833 ± 0.2	10.082 ± 3.0	1.80
**50**	1.356 ± 0.2	8.096 ± 1.0	3.180 ± 0.1	11.191 ± 3.5	1.63
**60**	1.500 ± 0.2	8.955 ± 1.3	3.573 ± 0.1	12.423 ± 4.0	1.51
**70**	1.675 ± 0.3	10.000 ± 1.6	4.062 ± 0.3	13.925 ± 4.6	1.42
**80**	1.917 ± 0.4	11.450 ± 2.2	4.758 ± 0.6	16.009 ± 5.6	1.34
**90**	2.352 ± 0.5	14.054 ± 3.3	6.056 ± 1.3	19.760 ± 7.3	1.04

e

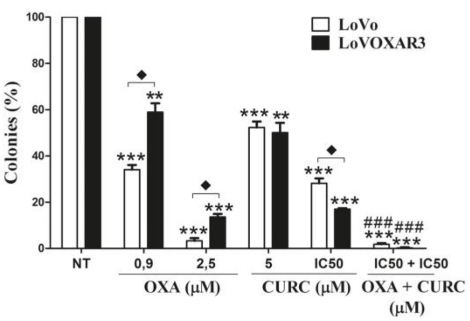

Supplementary Fig. S3. Combination of OXA and Curcumin in LoVO and LoVORAR3 cell lines. (a) Bar graphs representing mean ± SEM percentage of cell viability after a 24-hour treatment with OXA, Curcumin or their concomitant combination at the indicated doses in LoVo and **(b)** LoVOXAR3 cells. **(c)** OXA doses

(mean ± SEM) corresponding to indicated inhibitory concentrations (IC) in LoVo and LoVOXAR3 cells as a single agent or when combined with Curcumin concomitantly for 24 h. **(d)** OXA and Curcumin doses (mean ± SD) corresponding to the indicated inhibitory concentrations (IC) when given as single agents or in a 24h-concomitant schedule in LoVo (*top*) and LoVOXAR3 (*bottom*). CI represents the combination index values in each case. **(e)** Bar graph representing the percentage (mean ± SEM) of colonies in LoVo and LoVOXAR3 cells after 24 h of the indicated treatments. *p-value < 0.05; **p-value < 0.01; ***p-value < 0.001 relative to NT (Non-treated cells). #p-value < 0.05 relative to OXA individual treatment. ◆p-value < 0.05 as compared to LoVo.

Supplementary Figure S4

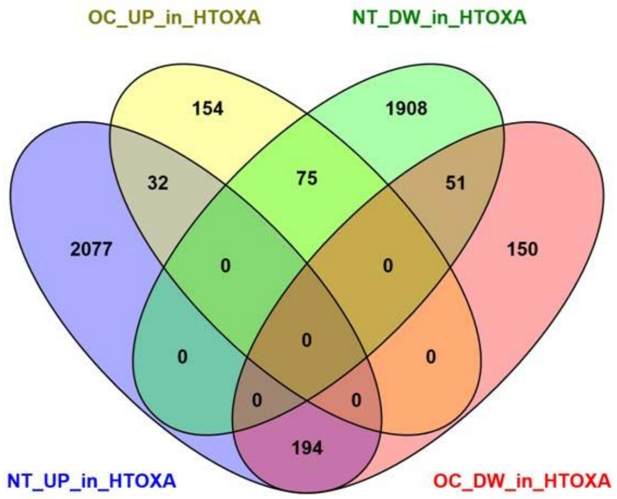

Supplementary Fig. S4. Venn diagram depicting the numbers of genes in the intersection between the the following conditions: OC_UP_in_HTOXA (genes with increased differential expression between oxaliplating plus curcumin treatment relative to untreated in HTOXAR3 compared to HT29), OC_DW_in_HTOXA (genes with decreased differential expression between oxaliplating plus curcumin treatment relative to untreated in HTOXAR3 compared to HT29), NT_UP_in_HTOXA HTOXA (genes upregulated in HTOXAR3 compared to HT29 under untreated basal conditions), and NT_DW_in_HTOXA (genes downregulated in HTOXAR3 compared to HT29 under untreated basal conditions).

Supplementary Figure S5

a

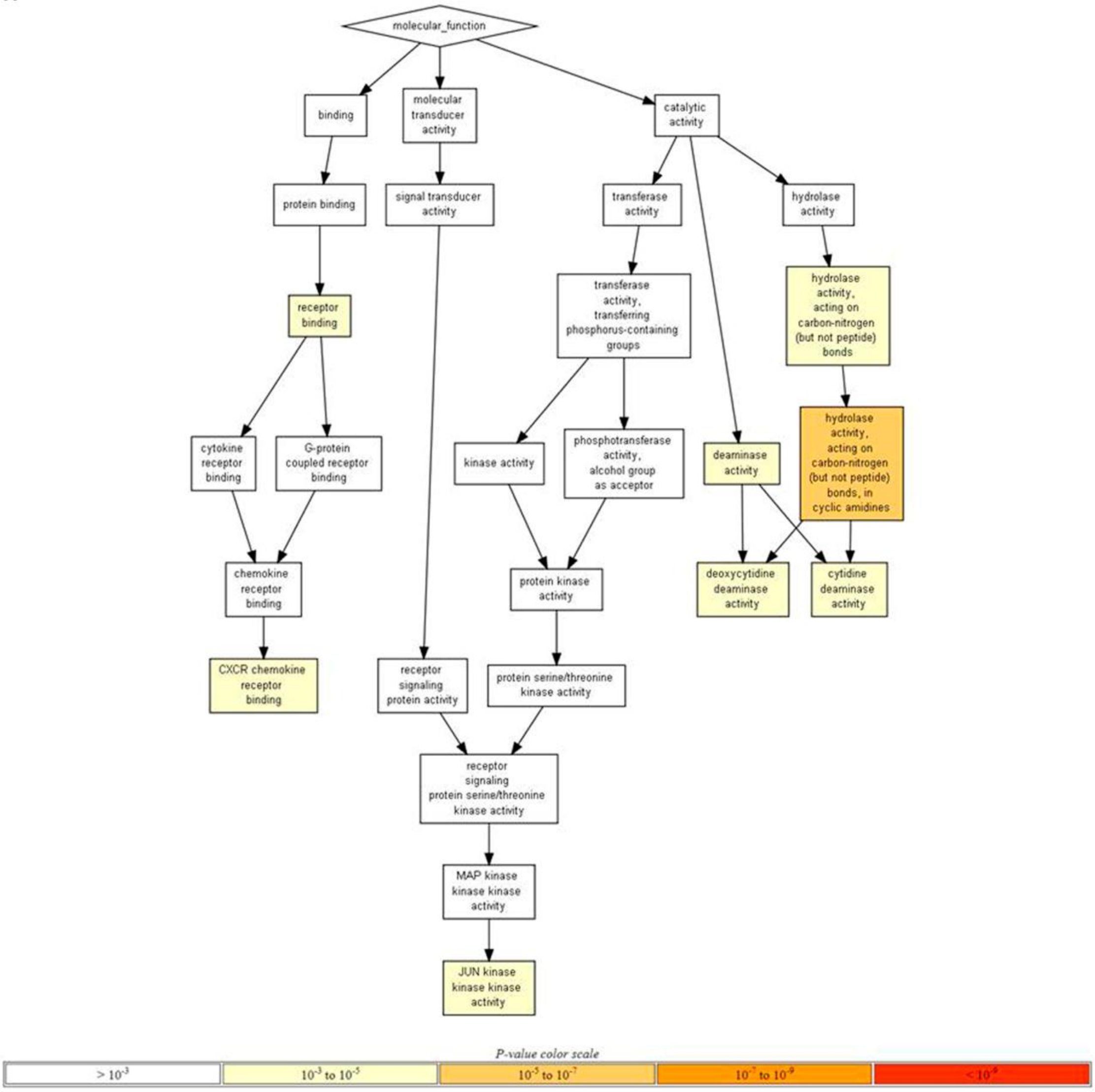

b

Gene Set Name	# Genes in Gene Set (K)	# Genes in Overlap (k)	k/K	p-value	FDR q-value
DEAMINASE_ACTIVITY	14	3	0.2143	1.49E-05	4.53E-03
HYDROLASE_ACTIVITY_ACTING_ON_CARBON_NITROGEN_NOT_PEPTIDEBONDSIN_CYCLIC_AMIDINES	16	3	0.1875	2.29E-05	4.53E-03
ISOMERASE_ACTIVITY	35	3	0.0857	2.54E-04	2.52E-02
RECEPTOR_BINDING	377	7	0.0186	3.91E-04	2.52E-02
CHEMOKINE_ACTIVITY	42	3	0.0714	4.38E-04	2.52E-02
CHEMOKINE_RECEPTOR_BINDING	43	3	0.0698	4.70E-04	2.52E-02
TRANSMEMBRANE_RECEPTOR_PROTEIN_TYROSINE_KINASE_ACTIVITY	43	3	0.0698	4.70E-04	2.52E-02
SIALYLTRANSFERASE_ACTIVITY	10	2	0.2	5.39E-04	2.52E-02
HYDROLASE_ACTIVITY_ACTING_ON_CARBON_NITROGEN_NOT_PEPTIDEBONDS	46	3	0.0652	5.74E-04	2.52E-02
CYTOKINE_ACTIVITY	113	4	0.0354	6.94E-04	2.75E-02
TRANSMEMBRANE_RECEPTOR_PROTEIN_KINASE_ACTIVITY	51	3	0.0588	7.77E-04	2.80E-02
G_PROTEIN_COUPLED_RECEPTOR_BINDING	54	3	0.0556	9.18E-04	3.03E-02
PROTEIN_TYROSINE_KINASE_ACTIVITY	63	3	0.0476	1.44E-03	4.38E-02
STRUCTURAL_MOLECULE_ACTIVITY	244	5	0.0205	1.75E-03	4.95E-02

Supplementary Fig. S5. Functional enrychment analysis. (a) Functional enrichment analysis of the 194 gene list up-regulated in HTOXAR3 at basal conditions and downregulated after OXA+Curcumin treatment **(b).** Significant GO molecular function terms enriched in the 194 gene list.

Supplementary Table S1

	Forward primer (5'-3')	Reverse primer (5'-3')
**CXCL8**	TCTTGGCAGCCTTCCTGATTTC	GTGTGGTCCACTCTCAATCACTCT
**CXCL1**	AACCCCAAGTTAGTTCAATCTGGA	CATGTTGCAGGCTCCTCAGAA
**CXCL2**	TCAAACCCAAGTTAGTTCAATCCTGA	GCTGACATGTGATATGTCATCACGAA

Supplementary Table S1. List of primers pairs used in RT-qPCR assay.

Supplementary Table S2

	Comparisons	total regulated genes	UP	DOWN
within cell line HT29	HT29__Oxaliplatin_vs_untreated	5726	2980	2746
	HT29__Oxaliplatin_plus_curcumin_vs_untreated	6622	3420	3202
	HT29__Oxaliplatin_plus_curcumin_vs_oxaliplatin	2447	1330	1117
within cell line HTOXA	HTOXA__Oxaliplatin_vs_untreated	4022	2095	1927
	HTOXA__Oxaliplatin_plus_curcumin_vs_untreated	6295	3194	3101
	HTOXA__Oxaliplatin_plus_curcumin_vs_oxaliplatin	5513	2766	2747
between cell lines HT29 and HTOXA	Untreated__HTOXA_vs_HT29	4337	2303	2034
	Oxaliplatin__HTOXA_vs_HT29	645	349	296
	Oxaliplatin_plus_curcumin_versus_basal__HTOXA_vs_HT29	656	261	395
	Oxaliplatin_plus_curcumin_vs_oxaliplatin__HTOXA_vs_HT29	165	48	117

Supplementary Table S2. Summary of regulated genes among 36,204 genes including unique gene symbols and other transcripts differentially expressed with the combined cut-off thresholds (|FC|>1.2 and q-value < 0.05)

Supplementary Table S3

Gene Set Name	# Genes in Gene Set (K)	# Genes in Overlap (k)	k/K	p-value	FDR q-value
HALLMARK_INTERFERON_GAMMA_RESPONSE	200	11	0.055	1.52E-10	7.58E-09
HALLMARK_COMPLEMENT	200	8	0.04	5.94E-07	7.43E-06
HALLMARK_EPITHELIAL_MESENCHYMAL_TRANSITION	200	8	0.04	5.94E-07	7.43E-06
HALLMARK_TNFA_SIGNALING_VIA_NFKB	200	8	0.04	5.94E-07	7.43E-06
HALLMARK_INTERFERON_ALPHA_RESPONSE	97	6	0.0619	1.28E-06	1.28E-05
HALLMARK_APOPTOSIS	161	6	0.0373	2.37E-05	1.97E-04
HALLMARK_HYPOXIA	200	6	0.03	7.93E-05	4.95E-04
HALLMARK_KRAS_SIGNALING_UP	200	6	0.03	7.93E-05	4.95E-04
HALLMARK_COAGULATION	138	5	0.0362	1.32E-04	7.34E-04
HALLMARK_ADIPOGENESIS	200	5	0.025	7.26E-04	2.59E-03
HALLMARK_ESTROGEN_RESPONSE_LATE	200	5	0.025	7.26E-04	2.59E-03
HALLMARK_MYOGENESIS	200	5	0.025	7.26E-04	2.59E-03
HALLMARK_P53_PATHWAY	200	5	0.025	7.26E-04	2.59E-03
HALLMARK_XENOBIOTIC_METABOLISM	200	5	0.025	7.26E-04	2.59E-03
HALLMARK_APICAL_JUNCTION	200	4	0.02	5.52E-03	1.53E-02
HALLMARK_ESTROGEN_RESPONSE_EARLY	200	4	0.02	5.52E-03	1.53E-02
HALLMARK_GLYCOLYSIS	200	4	0.02	5.52E-03	1.53E-02
HALLMARK_KRAS_SIGNALING_DN	200	4	0.02	5.52E-03	1.53E-02
HALLMARK_PEROXISOME	104	3	0.0288	5.93E-03	1.56E-02
HALLMARK_FATTY_ACID_METABOLISM	158	3	0.019	1.83E-02	4.36E-02
HALLMARK_UV_RESPONSE_UP	158	3	0.019	1.83E-02	4.36E-02

Gene Set Name	# Genes in Gene Set (K)	# Genes in Overlap (k)	k/K	p-value	FDR q-value
HALLMARK_TNFA_SIGNALING_VIA_NFKB	200	8	0.04	7.45E-10	3.72E-08
HALLMARK_P53_PATHWAY	200	5	0.025	1.33E-05	3.33E-04
HALLMARK_INFLAMMATORY_RESPONSE	200	4	0.02	2.41E-04	4.02E-03
HALLMARK_UV_RESPONSE_UP	158	3	0.019	1.77E-03	2.21E-02
HALLMARK_EPITHELIAL_MESENCHYMAL_TRANSITION	200	3	0.015	3.44E-03	2.87E-02
HALLMARK_KRAS_SIGNALING_UP	200	3	0.015	3.44E-03	2.87E-02
HALLMARK_CHOLESTEROL_HOMEOSTASIS	74	2	0.027	5.60E-03	4.00E-02

Supplementary Table S3. Significant Hallmark terms enriched in the 194 genes list repressed in resistant under basal conditions and activated by concomitant treatment (upper panel) and in the 75 gene list activated under basal conditions and repressed by combined treatment (lower panel) obtained by GSEA overlap.

Supplementary Table S4

GeneBank Accesion nº	Gene Name	Fold Change HTOXAR3 vs. HT29
**DNA REPAIR GENES**		
Poly (ADP-ribose) polymerase (PARP) enzymes		
NM_001618	PARP1	1.28
NM_001003931	PARP3	1.25
Repair of DNA-topoisomerase crosslinks		
NM_018319	TDP1	1.79
Mismatch excission repair (MMR)		
NM_002439	MSH3	1.27
NM_014381	MLH3	1.41
NM_000534	PMS1	-1.78
Nucleotide excission repair (NER)		
NM_004344	CETN2	1.22
NM_005316	GTF2H1	-1.25
NM_001517	GTF2H4	1.61
NM_001799	CDK7	1.30
NM_002431	MNAT1	1.55
NM_005236	ERCC4	1.27
Homologous Recombination		
NM_003579	RAD54L	1.34
NM_012415	RAD54B	-1.21
NM_182625	GEN1	-1.23
Fanconi Anemia		
NM_022725	FANCF	-1.48
NM_004629	FANCG	1.24
Non-homologous end-joining		
NM_021141	XRCC5	-1.23
Modulation of Nucleotide pools		
NM_015713	RRMB2	-1.24
DNA polymerases (catalytic subunits)		
NM_002912	REV3L	-1.27
NM_006502	POLH	1.23
Editing and procesing nucleases		
NM_033629	TREX1	1.22
NM_003686	EXO1	1.27
Ubiquitination and modification		
NM_152617	RNF8	1.22
Chromatin structure and modification		
NM_002105	H2AFX	-1.22
**DNA DAMAGE RESPONSE GENES**		
NM_001184	ATR	-1.20
NM_130384	ATRIP	1.26
NM_002853	RAD1	-1.26
NM_007194	CHEK2	-1.33

Supplementary Table S4. List of DNA repair and DNA damage response genes that are differentially expressed between OXA-sensitive and resistant cells ($|FC|>1.2$ and q-value < 0.05). Up-regulated DNA repair genes and down-reregulated DNA response genes in HTOXAR3 are highlighted in green.
